# Promoting STI testing among senior vocational students in Rotterdam, the Netherlands: effects of a cluster randomized study

**DOI:** 10.1186/1471-2458-11-937

**Published:** 2011-12-16

**Authors:** Mireille Wolfers, Gerjo Kok, Caspar Looman, Onno de Zwart, Johan Mackenbach

**Affiliations:** 1 Municipal Public Health Service Rotterdam-Rijnmond, Infectious Disease Control Division, P.O. Box 70032, 3000 LP Rotterdam, The Netherlands; 2 Department of Work and Social Psychology, Faculty of Psychology and Neuroscience, Maastricht University, Maastricht, The Netherlands; 3 Department of Public Health, Erasmus University Medical Centre, Rotterdam, The Netherlands; 4 Municipal Public Health Service Rotterdam-Rijnmond, Rotterdam, the Netherlands; 5 Department of Public Health, Erasmus University Medical Centre, Rotterdam, The Netherlands

## Abstract

**Background:**

Adolescents are a risk group for acquiring sexually transmitted infections (STIs). In the Netherlands, senior vocational school students are particular at risk. However, STI test rates among adolescents are low and interventions that promote testing are scarce. To enhance voluntary STI testing, an intervention was designed and evaluated in senior vocational schools. The intervention combined classroom health education with sexual health services at the school site. The purpose of this study was to assess the combined and single effects on STI testing of health education and school-based sexual health services.

**Methods:**

In a cluster-randomized study the intervention was evaluated in 24 schools, using three experimental conditions: 1) health education, 2) sexual health services; 3) both components; and a control group. STI testing was assessed by self reported behavior and registrations at regional sexual health services. Follow-up measurements were performed at 1, 3, and 6-9 months. Of 1302 students present at baseline, 739 (57%) completed at least 1 follow-up measurement, of these students 472 (64%) were sexually experienced, and considered to be susceptible for the intervention. Multi-level analyses were conducted. To perform analyses according to the principle of intention-to-treat, missing observations at follow-up on the outcome measure were imputed with multiple imputation techniques. Results were compared with the complete cases analysis.

**Results:**

Sexually experienced students that received the combined intervention of health education and sexual health services reported more STI testing (29%) than students in the control group (4%) (OR = 4.3, *p *< 0.05). Test rates in the group that received education or sexual health services only were 5.7% and 19.9%, not reaching statistical significance in multilevel analyses. Female students were more often tested then male students: 21.5% versus 5.4%. The STI-prevalence in the study group was low with 1.4%.

**Conclusions:**

Despite a low dose of intervention that was received by the students and a high attrition, we were able to show an intervention effect among sexually experienced students on STI testing. This study confirmed our hypothesis that offering health education to vocational students in combination with sexual health services at school sites is more effective in enhancing STI testing than offering services or education only.

## Background

Sexually transmitted infections (STIs) are serious health problems with 333 million new cases of curable STIs occur worldwide each year, with the highest rates among 20-24 year-olds, followed by 15-19 year-olds. Adolescents are believed to represent at least one third of cases of Chlamydia infection worldwide and perhaps an equal share of gonorrhea infection [1]. Also in the Netherlands heterosexually active adolescents are at high risk, with 50% of Chlamydia infections in 2009 occurring in those under 25 of years [2].

Effective prevention of STIs relies on condom use and on early case detection and treatment [3]; however, test rates among young heterosexual people are low. A Dutch national study in 2005 revealed that only 9% of boys and 14% of girls with sexual experience between 12 and 25 years had ever been tested for STI and 23% of boys and 26% of girls never used a condom with their last sex partner [4]. In the Netherlands, the general practitioner provides most STI-care [5]. Additional first line service is offered by specialized STI-centers, who provide free and anonymous STI care to high risk groups who do not want to visit their general practitioner for sexual health services, for example young people until 25 years [6].

Vocational students can be regarded as a high risk group for STIs. They become sexually active early, and STI prevalence seems to be higher than in their better educated counterparts. For example, 67% of the 16-19 year old students reported ever having vaginal sexual intercourse, compared with 51% of higher educated students [4]. Furthermore, Chlamydia rates are highest among low educated, urban heterosexual young people [7]. A Chlamydia positivity level of 24.5% was reported in a selective sample of vocational schools in our Municipal Public Health Service (MPHS) "Rotterdam-Rijnmond" in 2005 [8], with a similar rate in a repeated study at the same school in the subsequent year (unpublished data).

In the Netherlands, health services are normally not offered in schools. In an effort to reach high risk young people the MPHS is experimenting with offering sexual health services in senior vocational schools since 2008. In the curricula of most senior vocational school types sex-education is not included. However, the MPHS regularly received requests from schools to provide sexual health education. The experiences of the MPHS with outreach testing and health education [8] suggested a need for sexual health education combined with sexual health services. This is why the MPHS decided to develop a health education program.

The Dutch secondary school system comprises vocational schools and higher secondary schools (from 12 to 17-18 year). The vocational education includes two school levels: primary vocational schools (VMBO) for 12-16 year old children and senior vocational schools (MBO) for students from 16 years and above. The senior vocational school system provides courses at 4 levels: level 1 is the lowest level for which no former qualification is needed, level 2 provides education for carrying out work under supervision, while level 4 qualifies for an independent skilled job. According to the Dutch law on compulsory education children are obliged to go to school until their 18th birthday or until they have qualified for at least the 2nd level of senior vocational training. School fees are charged from the age of 18.

Approximately 50% of Dutch students attend a vocational school. In the high density urban areas many students are migrants or belong to minority ethnic groups. According to the level and educational attainment the ethnic background and gender of students vary substantially [9]. For example, technical courses are most often chosen by male students while courses in health and welfare are often chosen by female students with a non-Dutch ethnicity. The vocational schools in the urban areas in the Netherlands are known for a high dropout. As this is a possible limitation for an effect study, drop out was a matter of concern, while planning for evaluation. According to the schools, 10% study dropout was likely in the upcoming school year (2007-2008). Importantly, if the project started a few months after the beginning of the school year, much of this dropout would already have occurred (involving for example students who had never shown up and had thus been removed from the records, or those who had soon changed courses).

In the literature, most school-based sexual health interventions aim at decreasing sexual risk behavior by promoting abstinence, contraceptives, or condom use [10-13]. In the United States, the combination of school-based education and health services to improve sexual health is studied especially with respect to reproductive health services and in some cases with the provision of condoms [14,15], Health services can either be school-based or school-linked. School-based clinics are located on schools that offer services to students in their respective schools, while the school-linked clinics are adolescent clinics located near schools that provide many of the same services.

Hardly any studies report interventions to promote testing for STI. If they do, testing is described as a secondary outcome measure in a comprehensive program on sexual health [16,17].

Some programs offer STI screening to all students, for example in a study by Cohen [18] in which all students were offered a urine test on Chlamydia and Gonorrhea. All students were encouraged to participate regardless of whether they were sexually active.

We conducted a cluster randomized study to evaluate an intervention to promote STI testing among adolescents attending senior vocational schools. Our hypothesis is that combining health education with sexual health services at the school sites would enhance STI testing among senior vocational students.

## Methods

### The intervention

The intervention that we designed was called "ROsafe", and comprised two educational sessions, an Internet-based home-assignment and sexual health services on 4 afternoons or mornings at the school sites. The education on STI prevention and STI testing was embedded in 2 two-class period lessons on sexuality also covering basics of anatomy and physiology of the genitals, and contraceptives. The lessons were provided by a MPHS health educator or Public Health Nurse. The Internet site was to be visited as a home assignment for students to anonymously assess their personal risk for STIs and to motivate them to take an STI test if they had been involved in high-risk behaviour. The students were also advised on appropriate test locations: did they want to have an anonymous STI test, did they prefer to go to their general practitioner, or did they need a service that was free of charge. Furthermore, by taking the quiz on the Internet site, students could test and improve their knowledge on safe sex and STIs. It took approximately 25 min to complete the assignments. The intervention addressed the attitude, social norms, risk perception, self efficacy and accessibility of sexual health services, all behavioral determinants that were found to correlate with STI testing among this group of adolescents [19]. The theory- and evidence-based development of this intervention is described in more detail elsewhere [20].

The sexual health services were free of charge, anonymous, and without an appointment. The nurse was seeing students in an office or classroom situated at a quiet and private location within the school building. Service included testing for Chlamydia, Gonorrhea and HIV, but also advice concerning contraception, safe sex and sexual health in general. Urine samples were used for testing on Chlamydia and Gonorrhea. The students got their test results by SMS (text message). If the test turned out to be positive, they were invited to make an appointment for treatment at the health service.

### Study design

The intervention was evaluated using a cluster randomized study with three experimental conditions: group 1 received health education combined with sexual health services at the school site; group 2 received only health education, while group 3 received only sexual health services. The control group did not receive any of the intervention elements.

A baseline level of STI testing of 10% was assumed. In general, it was expected that students who were tested for STI at baseline, would be twice as likely to get tested than students who had not been tested at baseline. It was calculated that 6 schools per condition, including 80 students per school were needed to power the study to detect a difference in test rate in at-risk students between the intervention group and the control group of 12.5% (15% tested students in the intervention group versus 2, 5% in the control group) in those who were not tested before, and a difference of 25% in those who had previous been tested (30% tested students versus 5%) (with β = 0.80 and α = 0.05 in a two tailed test). Based on previous research [4,19] it was expected that 70% of the students would have sexual experience, and 50% of them would have had sexual intercourse with more than one partner. Students with multiple lifetime partners were expected to be at risk for STIs [7,21]. A loss to follow-up of 14% was accounted for. Schools were the unit of randomization; in each school 4-6 classes were randomly selected to participate in the intervention group in which the school was randomized to.

Student data were collected at baseline (2 weeks before the intervention), 1, 3, and 6-9 months after the intervention. After the final measurement, classes in group 3 and 4 were offered the health education in the next school year. Approval for this study was provided by the ethics committee of Erasmus Medical Center.

### Participants

To be eligible for the study, schools had to offer fulltime courses to adolescent students, and have at least four classes available with a curriculum lasting for another 18 months. Of 39 schools, 28 schools were eligible for the study: nine schools could not provide four classes with long term courses, and two were involved in a Healthy School pilot, which might disturb the experimental condition. 24 schools were randomly selected from the sample of 28 eligible schools. One school refused to participate after randomization was done, due to organizational changes and was replaced by another school. During the study period it appeared that in two control schools not enough classes participated, due to a lack of collaboration of the teachers. To replace these classes we recruited 4 extra classes at the other participating schools.

The baseline questionnaire was completed by 1361 students. However, 1903 students completed questionnaires after baseline, indicating that more students were involved at the follow-up questionnaires than there were at baseline. Because of missing values on matching variables, 59 baseline questionnaires and 96 follow-up questionnaires were removed. Only students with sexual experience were considered to be susceptible for the intervention (uptake of an STI test). Of the 1302 students who were present at baseline, 822 (63%) had experience with sexual intercourse and were eligible for intention-to-treat analysis. Of the total group of 1302 students with a baseline measurement, for 739 (57%) students at least one follow-up questionnaire could be correctly matched.

**Figure 1:**
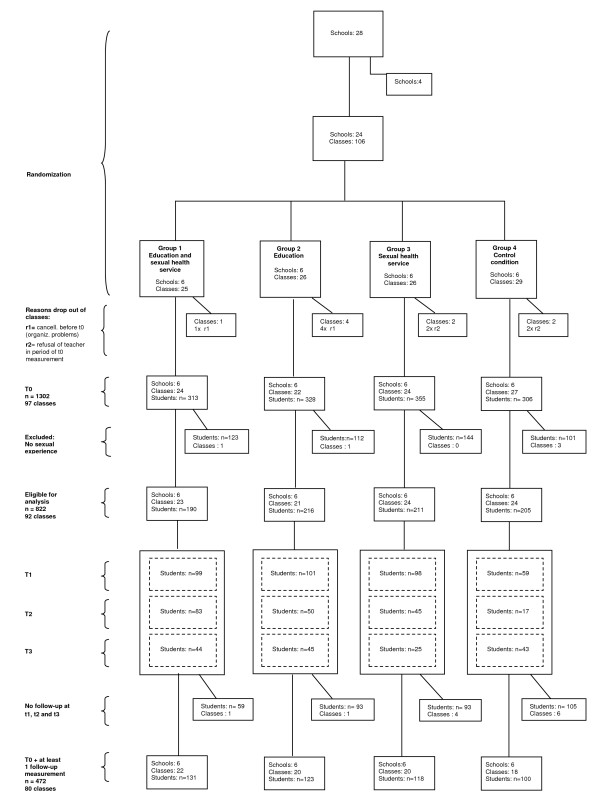
**Participants flow and drop out**.

The final study sample of students consisted of 80 classes in 24 schools and 472 sexually experienced students whose baseline and at least one follow-up questionnaire could be correctly matched. Matching criteria were 1) school, 2) class, 3) date of birth, 4) postal code. Unavailability at baseline or follow-up was primarily due to absentee-ism, transfer to other classes or schools, or missing data on matching variables. Sometimes teachers were not cooperative in administering each follow-up questionnaire to their students; heavy workload, illness of participating teachers, cancellation of lessons, exams, and periods of work placements when students were not in school were reasons why questionnaires were not filled out. See Figure 1 for participants flow and drop out.

### Procedure

The evaluation was carried out in the classrooms by filling out pen and paper questionnaires. Administration was supervised by the teacher and sometimes by a member from the research team as well. Students were asked to put their questionnaire in a sealed envelope, and put it in a sealed mailbox. The box was collected by the researchers. Variation in interval between second and third follow-up measurement was caused by the summer holidays. Students were not given any incentive to attend intervention activities. When completing the questionnaires they received only a pen, a condom and a small gadget worth approximately 1.50 $.

At the sexual health services at the school, registrations were kept on the visits. If requested by the student, this information was kept anonymous, with a nickname and mobile phone number only to report their test results. The three sexual health services for young people in the area as well as the local STI clinic asked their adolescent clients if they attended one of the participating schools and participated in the project. Students who visited one of the health services were asked written permission to match the files of their visit to the questionnaire they filled out in the classroom.

### Measures

#### Testing behavior

At baseline, students had to complete questions regarding their history of STI testing. At 1st, 2nd and 3rd follow-up assessment, they were again asked whether they had been tested for STI in the past month, in the past 2 months, or since the beginning of the year, while specifying the time of the test (the intervention started in January of the same year). The answers of the three follow-up questionnaires were recorded into one outcome measure for STI testing after the intervention.

#### Sexual behavior

At baseline assessment, students had to respond to several questions on their relationships and experience with sexual intercourse (anal and vaginal intercourse). Students who had experience with sexual intercourse were also asked to respond to items on condom use over the past 2 months and in the most recent sexual anal and/or vaginal intercourse. Condom use during vaginal and anal sex in both steady and casual relationships was measured using a five-point scale ranging from 'never' to 'always'. All items were derived from a Dutch national study on sexual behavior among adolescents [4].

A sexual behavior risk index ranging from 0 (no risk), 1 (low risk) and 2 (high risk) was created. No risk was defined by abstinence. Low risk was assigned to participants that consistently used a condom in vaginal or anal intercourse in the previous 2 months and who reported having used a condom in the most recent intercourse. Participants that did not report sex in the previous 2 months but did report having used a condom in the most recent intercourse were also considered low risk. Lastly, participants that reported inconsistent condom use over the previous 2 months were classified as high risk, as were participants that did not report sexual intercourse in the previous 2 months but did report not having used a condom in the most recent intercourse.

#### Demographics

Students had to complete questions regarding their age, gender, ethnic background, religious affiliation, class, school, type and level of vocational course.

#### Process measures

In the first follow-up questionnaire items were added to assess the reach of the intervention and the dose received. Students were asked whether they had attended the lessons, whether they had visited the projects' website for the home assignment, whether they knew about the sexual health services at their school and if they had used these services. Students were also asked to rate their appreciation of the lessons at a scale from 1 to 10 (10 representing the highest score), whether they considered that they had learned something new and if the topics taught seemed personally relevant to them (both questions were asked on a scale from 1 to 5) (not agree at all, to totally agree). The implementation of the lessons was monitored using a checklist for the educators to complete after each lesson, to see whether the lessons were executed completely. Two lessons of each health educator were observed by the researcher.

### Data analysis

Missing value analyses were performed for students lost to follow-up as compared to students with one or more follow-up measurements, using Chi-square tests and Kruskal Wallis tests (using SPSS 15.0) for comparisons on socio-demographic characteristics (gender, ethnicity) and experience with sexual intercourse and relationships.

In the eligible sample for analysis (n = 822, Figure 1), 43% of respondents were absent at follow-up. To perform analyses according to the principle of intention- to- treat, missing observations at follow-up on the outcome measure of being tested on STI were imputed with multiple imputation techniques, using nearest neighbor hot deck procedure [22,23] in R version 2.7., using 23 covariates. Mahalanobis distance was used to calculate the similarity between units. Results based on complete cases analysis (n = 472) were compared with results based on the 822 cases sample, after multiple imputation was performed.

For the evaluation of the effectiveness of the intervention we compared each of the experimental conditions with the control condition, using multilevel analysis to allow for the clustering of observations within schools and within classes, using ML-win version 2.02 [24]. Descriptive analyses comparing conditions were conducted to check whether the randomization had resulted in a balanced distribution of important characteristics of the students. Most schools offer courses in a certain sector that is specifically chosen by either boys or girls, resulting in health and welfare classes with predominantly female students or technique classes with only male students. Therefore the cluster randomization of a relatively small number of schools leads to imbalance in the percentage of female students.

To determine whether the demographic characteristics sex, age, ethnicity and test-history, should be used as covariates, univariate logistic regression analyses were performed to assess whether they were related to testing behavior at follow up. Sex and test-history were important predictors of testing: females were 6 times more likely to be tested than males, also being tested in the past predicted testing (OR 3.5). These three variables were used as covariates in the multilevel model. Informed by previous research [7], ethnicity was also added as a covariate. By using multilevel analysis variances within and between schools and classes can be estimated and corrected for. The multilevel analyses showed that the intercept was random for class level, but not for school level. This means that the characteristics of the population of students belonging to a particular class differ from those of another class, caused by certain characteristics or circumstances shared by students in the same class. It was therefore necessary to correct for class in the regression analysis, this means that different intercepts are estimated for each class [24].

## Results

### Baseline characteristics

Table 1 shows demographic variables and baseline measurements for experience with relationships and sexual intercourse of students in the intervention groups and the control group. Sixty percent of the sample was female and had a Dutch ethnic background, with an average age of 18.2 years. Half of the students were involved in a steady relationship and 64% had experience in vaginal intercourse. Seventy percent had had sexual intercourse with more than 1 partner, while 11% had sex with more than 1 partner in the past 2 months. Baseline characteristics did not differ on age, sex and ethnicity, experience with relationships, sexual behavior or condom use. As a consequence of the study design with schools as the unit of randomization, educational attainment differed between the groups.

**Table 1:** Baseline characteristics of students according to experimental group (n = 739)

	Total group			Intervention group 1: Education and sexual health service		Intervention group 2: Education		Intervention group 3: Sexual health service		Control group		*p*-value*	Chi-square (df)
	n			n = 213		n = 196		n = 183		n = 147			
Age, average (SD)	739	18.2	(2.25)	18.5	(2.75)	17.8	(1.74)	18.1	(2.33)	18.2	(1.89)	0.15	1.76(3)^e^
Sex												0.37	3.11(3)
Male	739	298	40.3%	62	29.1%	87	44.4%	57	31.1%	92	62.2%		
Female	739	441	59.7%	151	70.9%	109	55.6%	126	68.9%	55	37.4%		
Ethnicity	739											0.27	3.90(3)
Dutch		446	60.4%	112	52.6%	117	59.7%	114	62.3%	103	70.1%		
Non-Dutch		293	39.6%	101	47.4%	79	40.3%	69	37.7%	44	29.9%		
Type of education	739											NA	
Health &welfare		357	48.3%	165	77.5%	56	28.6%	79	43.2%	57	38.8%		
Economic		286	38.7%	47	22.1%	108	55.1%	104	56.8%	27	18.4%		
Technical		96	13.0%	1	0.5%	32	16.3%	0	0%	63	42.9%		
Has a steady relationship	732	372	50.8%	110	52.1%	96	49.7%	100	55.2%	66	44.9%	0.97	0.24(3)
Experience with vaginal intercourse	739	472	63.9%	131	61.5%	123	62.8%	118	64.5%	100	68.0%	0.99	0.098(3)
Experience with anal intercourse	739	73	9.9%	17	8.0%	24	12.3%	14	7.7%	18	12.2%	0.25	4.10(3)
Number of lifetime partners vaginal intercourse	420^a^											0.12	14.17(9)
1		129	30.7%	33	31.1%	36	31.0%	40	37.0%	20	22.2%		
2		76	18.1%	24	22.6%	22	19.0%	17	15.7%	13	14.4%		
3-5		138	32.9%	33	31, 1%	34	29.3%	37	34.3%	34	37.8%		
6+		77	18.3%	16	15, 1%	24	20.7%	14	13.0%	23	25.6%		
Number of partners vaginal intercourse in past 2 months	428^b^											0.59	4.63(6)
0		102	23.8%	32	29.6%	25	21.0%	21	19.3%	24	26.1%		
1		279	65.2%	64	59.3%	80	67.2%	79	72.5%	56	60.9%		
2+		47	11.0%	12	11.1%	14	11.8%	9	8.3%	12	13.0%		
Condom use in last vaginal intercourse	444^c^											0.82	0.90(3)
Yes		230	51.8%	48	43.2%	64	52.5%	52	46.8%	50	50%		
No		214	48.2%	63	56.8%	58	47.5%	59	53.2%	50	50%		
Consistent condom use in past 2 months	292^d^											0.76	1.16(3)
Yes		189	64.7	17	29.3%	27	31.8%	26	33.8%	33	47.1%		
No		103	35.3	43	71.7%	58	68.2%	51	66.2%	37	52.9%		
Experience with STI testing	719	62	8.6%	23	11.0%	15	8.1%	17	9.4%	7	4.9%	< 0.001	487(3)

* Real numbers and percentage are reported in this table, but p-values for differences between the groups were tested using multilevel and multinominal regression models with random effects to account for the clustering of students within classes

^a ^Selection of persons with experience in vaginal intercourse (n = 472), number of partners is missing for 52 cases (11.0%)

^b ^Selection of persons with experience in vaginal intercourse (n = 472), number of partners is missing for 44 cases (9.3%)

^c ^Selection of persons with experience in vaginal intercourse (n = 472), number of partners is missing for 28 cases (5.9%)

^d ^Selection of persons who had vaginal intercourse

^e ^F-statistic

Students who dropped out after the baseline questionnaire were more often male (48%) than students that remained in the study (40, 3%; *χ*^2 ^(1, N = 1302) = 8.28; *p *< 0.01) and more often had a migrant background (52%) than those who were retained for follow-up (60%; *χ*^2 ^(1, N = 1302) = 18.73, *p *< 0.001) (Table 2). Drop outs also reported more lifetime partners with whom they had vaginal intercourse: 30% reported 6 or more partners, compared to 18% (*χ*^2 ^(1, N = 731) = 9.55, *p *< 0.05) reported by students that remained in the study.

**Table 2:** Characteristics of students who were retained in the study and those who were lost to follow-up (n = 1302)

		Students with follow-up		Students lost to follow-up		*p*-value
		**N**	**%**	**N**	**%**	
Sex						
Male^a^		298	40.3%	272	48.3%	< 0.01
Female		441	59.7%	291	51.7%	
Ethnicity^a^						
Dutch		446	60.4%	272	48.3%	< 0.001
Non-Dutch		293	39.6%	291	51.7%	
Has a steady relationship^a^						
Yes		372	50.8%	265	48.8%	0.26
No		360	49.2%	278	51.2%	
Experience with vaginal intercourse^a^						
Yes		472	63.9%	350	62.3%	0.56
No		267	36.1%	212	37.7%	
Experience with anal intercourse^a^						
Yes		73	9.9%	68	12.2%	0.21
No		665	90.1%	490	87.8%	
*Subgroup of students with experience in vaginal intercourse: *						
	Total n of subsample					
Number of lifetime partners vaginal intercourse^b^	731^c^	n = 731				
1		129	30.7%	79	25.4%	< 0.05
2		46	18.1%	47	15.1%	
3-5		138	32.9%	88	28.3%	
6+		77	18.3%	92	29.6%	
Number of partners vaginal intercourse in past 2 months^b ^n = 755	755^d^					0.46
0		102	23.8%	79	24.2%	
1		279	65.2%	191	58.4%	
2+		47	11.0%	57	17.4%	
Condom use in last vaginal intercourse^a^						
Yes		214	48.2%	152	45.5%	0.25
No		230	51.8%	182	54.5%	
Consistent condom use in past 2 months^a^						
Yes		103	35.3%	79	33.9%	0.41
No		189	64.7%	154	66.1%	

^a ^Tested using the Chi Square test

^b ^Tested using the Kruskal Wallis test

^c ^Selection of persons with experience in vaginal intercourse (n = 822), number of partners is missing for 91 cases (11.1%)

^d ^Selection of persons with experience in vaginal intercourse (n = 822), number of partners is missing for 67 cases (8.2%)

### Process measures

The implementation of the lessons was monitored with a checklist for the educators to complete after each lesson: the five health educators had performed all activities in the protocol. In 5 classes they were not able to provide both lessons, twice this was due to organizational problems, and on 3 occasions, students did not turn up for the second lesson. In the intervention groups 1 and 2 (the groups that received the educational intervention), 187 (74%) of the sexually experienced students (n = 254) filled out the first follow-up questionnaire. Of those, 164 students (88%) had attended at least one lesson, 63% attended both lessons, 44% performed the Internet assignment and 32% received the full educational intervention which comprised both lessons plus the Internet application. The lessons were highly appreciated by the students, they rated it 8.3 (scale from 1 to 10); 51% of the students indicated that they had learned something new during the lessons and 68% qualified the topics in the lessons as personally relevant. The intervention dose received did not differ significantly by intervention group.

### Effects on STI testing

Table 3 shows the number of sexually experienced students in the intervention groups and the control group that reported an STI test after the intervention. With 29% of the students who performed an STI test after the intervention, intervention group 1 reported the highest number of STI tests at follow-up. Female students were more often tested than male students: 21.5% versus 5.4%.

**Table 3:** Persons tested at follow-up (students with experience in sexual intercourse)

	Intervention group 1: Education and sexual health service			Intervention group 2: Education			Intervention group 3: Sexual health service			Control group			Total group		
	**n**	**N**	**%**	**n**	**N**	**%**	**n**	**N**	**%**	**n**	**N**	**%**	**n**	**N**	**%**
Tested on STI after intervention	38	131	29.0%	7	123	5.7%	20	118	16.9%	4	100	4.0%	69	472	14.6%
Males	4	38	10.5%	1	56	1.8%	3	41	7.3%	3	67	4.5%	11	202	5.4%
Females	34	93	36.6%	6	67	9.0%	17	77	22.1%	1	33	3.0%	58	270	21.5%

Table 4 reports the different test venues and test results of the students who reported an STI test. It reveals that, in the groups that were offered sexual health services at the school, most students reported to have done the test at their school (Table 4). Only 1 student reported to be tested positive for STI.

**Table 4:** Diagnosis and test location of students who are tested for STI at follow-up

	Intervention group 1 Education and sexual health services		Intervention group 2 Education		Intervention group 3 Sexual health services		Control group		total	
	**n = 38**		**n = 7**		**n = 20**		**n = 4**		**n = 69**	
Positive diagnosis	0	0%	1^a^	14.3%	0	0%	0	0%	1	1.4%
Test location:										
School-service	27/38	71.1%	0	0%	17/20	85.0%	0	0%	44/69	63.8%
General practitioner	7/38	18.4%	2/7	28.6%	5/20	25.0%	3/4	75.0%	17/69	24.6%
Other location	7/38	18.4%	4/7	57.1%	1/20	5.0%	1/4	25.0%	11/69	15.9%

^a ^Tested positive for Chlamydia

^b ^Total can add up to > 100%: some participants reported tests in more than one location

From the total of 69 self reported STI tests, 42 (61%) could be confirmed by the registrations. Registrations were kept at the sexual health services at the school sites, at the three regular sexual health services for young people in the area, and at the local STI clinic. For those 42 students the information on their identity in the questionnaire could be linked to the services at which they were tested. However, in the registrations of the school sexual health services for 50% of the students their postal code or date of birth was missing. These were needed to link their test results to their questionnaire.

Test results of multilevel analyses are reported in Table 5 and 6. The complete cases analysis for this group of students (n = 472) showed a positive intervention effect for intervention group 1 (OR = 4.3, *p *< 0.05), adjusted for test-history at baseline, sex and ethnicity. To see whether there was a difference in intervention effect between students with a high and low risk score, we added the sexual behavior risk index score to the model and performed additional analyses with interactions between intervention group and sexual risk index. There was no significant effect of either the risk score or the interaction with the intervention group, indicating that high risk students in the intervention group 1 were not more frequently tested than low risk students. The intention-to-treat analysis (n = 822) showed a similar effect for intervention group 1 as the complete cases analyses (OR 3.6; *p *= 0.02). The intraclass correlation coefficient (ICC) of .28 for class level, indicates that there was a high degree of similarity between students within the same class.

**Table 5:** Results of multilevel analysis to assess intervention effects on STI testing: complete cases analysis (n = 472)

	Model 1: random intercept for class, with individual variables and baseline			Model 2: Model 1 with intervention conditions		
Measures of variation of clustering:						
Class level variance (SE)	1.72(0.56)			1.25(0.49)		
ICC Class level	0.34			0.28		
						
Individual level variables:						
	Beta (SE)	OR (95%CI)	*p*-value	Beta	OR (95%CI)	*p*-value
Sex (female vs male)	1.35(0.41)	3.86 (1.72-8.66)	< 0.01	1.05 (0.42)	2.84 (1.24-6.46)	< 0.05
Ethnicity (Dutch vs non-Dutch)	-0.43(0.37)	0.65 (0.29-1.45)	0.29	-0.50 (0.37)	0.61 (0.30-1.25)	0.17
Baseline test behavior	1.16(0.36)	3.18 (1.57-6.45)	< 0.001	1.24 (0.37)	3.46 (1.66-7.19)	< 0.001
Intervention group 1^a^				1.46 (0.71)	4.25 (1.07-17.25)	< 0.05
Intervention group 2^b^				-0.06 (0.78)	0.94 (0.21-4.31)	0.92
Intervention group 3^c^				0.96 (0.71)	1.70 (0.64-10.56)	0.18

^a ^= educational intervention and sexual health service, ^b ^= educational intervention, ^c ^= sexual health service

**Table 6:** Results of multilevel analysis to assess intervention effects on STI testing: imputed data for missing cases at follow-up (n = 822)

	Model 1: random intercept for class, with individual variables and baseline		Model 2: Model 1 with intervention conditions			
Measures of variation or clustering:						
Class level variance	1.29			0.82		
ICC Class level	0.28			0.20		
Individual level variables:						
	Beta (SE)	OR (95%CI)	*p*-value	Beta	OR (95%CI)	*p*-value
Sex (female vs male)	0.90 (0.33)	2.46 (1.29-4.70)	0.01	0.70 (0.32)	2.02 (1.08-3.80)	0.03
Ethnicity (Dutch vs non-Dutch)	0.26 (0.29)	1.29 (0.74-2.26)	0.37	0.33 (0.28)	1.39 (0.80-2.42)	0.24
Baseline test behaviour	0.90 (0.39)	2.46 (1.15-5.28)	0.02	-0.88 (0.39)	2.43 (1.13-5.22)	0.02
Intervention group 1^a^				1.29 (0.54)	3.62 (1.26-10.40)	0.02
Intervention group 2^b^				-0.26 (0.66)	0.77 (0.21-2.80)	0.69
Intervention group 3^c^				0.71 (0.52)	2.04 (0.74-5.64)	0.17

^a ^= educational intervention and sexual health service, ^b ^= educational intervention, ^c ^= sexual health service

## Discussion and conclusion

### Discussion

The "ROsafe" program was designed as an intervention with an educational as well as a service providing component, based on evidence and theory to increase STI testing behavior by senior vocational students. The results of this cluster randomized study suggest that the intervention that offered education combined with health services had positive effects on the uptake of STI testing among students with sexual experience. Compared to a control group, students that received the health education and were offered sexual health services in the schools were more often tested (OR 4.25) for STI than students who received only one of these components.

The prevalence rate of STI of students in the study group tested after the intervention was lower than expected. Only 1.4% of the sample tested positive for Chlamydia, while in a large national Chlamydia study in the Netherlands an overall prevalence of 2.0% was found, with 3.2% in highly urban areas (in which our study group lives) [7]. Furthermore, in higher risk populations such as in visitors of the Rotterdam STI clinic, Chlamydia prevalence is around 10% [8].

The hypothesis that offering school based facilities can enhance the uptake of sexual health care is supported by other studies [25-29]. Characteristics of the services provided in this study are in line with those that are identified as facilitators for the use of school based sexual health services for adolescents [10]. For example, the services were offered in a space that was confidential, easy accessible, free of charge, with the possibility to take a friend along. Our study is rather unique because, to our knowledge, no studies exist on school-based interventions to promote STI testing among adolescents that combine health education and sexual health services. However, school-based interventions aiming at testing (among other outcomes) comprising only educational components could not discover significant effects on STI testing [16,17].

Female students were more often tested than male students. The difference in test-rate between female students and male students was large: 21.5% of sexually experienced women versus 5.4% of the men performed an STI test. Also at the school based sexual health service most of the clients were female. Perhaps school based health services are more accepted by women, as was also suggested in a review by Kirby who observed that older males may not be easily reached by school- based or school-linked clinics in the United States [14]. European studies also suggest that male students have other preferences than female students with respect to school based health services and school based sexual health services. E.g. a Swedish study by Makenzius among male students revealed that a majority of them felt the need for counseling and advice about their sexuality, but that they needed more male-friendly youth health services, such as male staff, special hours for males and alternative methods of STI testing, such as Internet-service for Chlamydia testing [30]. The preference for male staff by boys was also reported in a study among British secondary school students. The school drop-in clinics were most frequently visited by girls [31]. However, also among the general STI clinic visitors (not school- based) below 25 years in the Netherlands, 65% is female [32,33].

Students who were absent at baseline were excluded from the analysis. However, in the entire sample which also includes those without a baseline measurement (n = 1762), reported STI prevalence was 8.0%. This indicates that students that were tested for STI during the time of the intervention were more often absent at baseline. Possibly, these students were more at risk for STI than those who were present al baseline. Data on their sexual behavior is available from the 2 months preceding their follow up measurement, and shows that their sexual behavior was not riskier from students who were compliant at baseline. However, evidence exist that students who drop out or have less attachment to school have more sexual risk behavior due to a riskier lifestyle which might be related to absenteeism and drop out [14].

Several methodological limitations should be considered in interpreting this study. Although teachers were requested to administer questionnaires to absent students in one of the following lessons, this study was confronted with a high attrition. Only 57% of the baseline sample was compliant at one or more follow-up measurements, due to high levels of absenteeism from school among these students. It is unknown whether this absenteeism is in any way related to the study, because the participating schools could not provide figures on absenteeism at school or class level. However, the Ministry of Education calculates school drop-out rates on the base of registrations at the start and finish of the school year. The 2009 figures showed a 13% drop out at vocational schools in the Rotterdam-Rijnmond area in the school year 2007/2008 [34].

Another reason for loss to follow-up was transfer of students to another class or school. Also, not all teachers were cooperative in administering each follow up questionnaire to their students, due to workloads, illness, cancellations of lessons, exams, and periods of work placement when students were not in the school. The cooperation of teachers also depended on their degree of involvement with the subject. High attrition rates for intervention research or low compliance in a school screening program are not rare in this type of research in schools, especially when targeting high risk youth [16,35,36]. The outcome measure of STI testing could be constructed if a student was compliant for at least 1 follow-up measurement. Due to missing data at follow-up measurements, test rates can be underestimated. If a student reported no test at t1, but dropped out after t1, a possible test after t1 is not reported. Self reports were the principal mean of data collection. Where possible, client registrations of the health services were matched to the self reports. However, only 61% of self reported STI tests could be successfully matched. Apart from the low response rates at the follow-up questionnaires this was due to the fact that students could choose to be seen anonymously, and at the questionnaires only a few identifying questions were asked (not their name).

A second limitation, also caused by a high degree of absenteeism, was that only a minority of the students received the complete intervention: only 32% of students who filled out the questionnaire at t1 reported to have received the full intervention, However, 26% did not fill out the t1 questionnaire, and it is unknown whether they received any of the intervention components.

## Conclusion

Despite a low dose of intervention that was received by the students and a high attrition, we were able to show an intervention effect among sexually experienced students on STI testing. This study confirmed our hypothesis that offering health education to vocational students in combination with sexual health services at school sites is more effective in enhancing STI testing than offering services or education only. It will be interesting repeating this type of research in a school setting with lower drop-out and absenteeism rates. Also, alternative study designs should be considered to evaluate interventions in school settings with a large drop out, while senior vocational students are an important target group.

## Competing interests

The authors declare that they have no competing interests.

## Authors' contributions

MW conceived of the study, participated in its design, carried out the study, performed the analyses and drafted the manuscript. GK discussed interpretation of results and provided comments on the manuscript. CL performed the power calculation, the multiple imputation and helped performing the multi-level analyses; JM helped conceive the study, participated in the design of the study and provided comments on the manuscript. OZ participated in the design of the study, helped draft the manuscript and coordinated the study. All authors read and approved of the final manuscript.

## Pre-publication history

The pre-publication history for this paper can be accessed here:

http://www.biomedcentral.com/1471-2458/11/937/prepub
